# Cold bonding of aluminium to copper by deformation-enhanced diffusion

**DOI:** 10.1038/s41598-025-25620-1

**Published:** 2025-11-24

**Authors:** Per Erik Vullum, Ambra Celotto, Øystein Grong, Randi Holmestad

**Affiliations:** 1 SINTEF Industry, 7034 Trondheim, Norway; 2 Department of Physics, Norwegian University of Science and Technology (NTNU), 7491 Trondheim, Norway; 3 Department of Mechanical and Industrial Engineering, Norwegian University of Science and Technology (NTNU), 7491 Trondheim, Norway

**Keywords:** Engineering, Materials science, Physics

## Abstract

Cold bonding between dissimilar metals and alloys offers several benefits, such as the absence of soft heat-affected zones and thick intermetallic layers with properties that differ from those of the base metals. At room temperature, thermally driven diffusion processes are negligible, but we demonstrate here that mechanically strong bonds are still feasible through high plastic deformation and high strain rates. If the strain rate is high and the temperature is kept low to avoid significant annihilation of vacancies, mechanically strong bonds involving intermetallic compound formation can still form through vacancy-driven diffusion across the contact interface. In the present investigation, two different experimental setups were employed to demonstrate strong bonding between copper and aluminium at room temperature, using phenomenological equations for excess vacancy formation and diffusion to highlight the underlying bonding mechanisms involved. Transmission electron microscopy is used to verify the bonding mechanisms.

**Supplementary Information:**

The online version contains supplementary material available at 10.1038/s41598-025-25620-1.

## Introduction

In an industrial context the term “solid-state joining” covers several different joining processes, including diffusion welding, explosion welding, forge welding, conventional friction welding, friction stir welding, hybrid metal extrusion & bonding, hot pressure welding, ultrasonic welding and cold pressure welding^1,2^. These processes offer the advantage of enabling bonding at temperatures below the melting point of the metal parts to be joined, generally without the use of a brazing filler metal. Hence, in the as-welded condition, the base metal will largely retain its microstructural integrity without forming a fusion zone and a wide heat-affected zone (HAZ) with degraded properties^3–5^. In the case of dissimilar metals, the lack of compatibility in chemical composition, melting temperature, crystal structure, thermal expansion and conductivity between the two components to be joined makes fusion welding particularly challenging^6,7^. Therefore, for most metal–metal combinations, solid-state joining is the best choice, as it reduces the risk of excessive intermetallic compound (IMC) formation and subsequent interfacial cracking^8–18^.

Of the different solid-state joining techniques listed above, only cold pressure welding (CPW) can be classified as a “cold bonding” method. In the present investigation, cold bonding refers to joining carried out at room temperature (RT) by plastic deformation, without the use of external Joule heating or frictional heating. This technique was employed in ancient times by the Greek Mycenaean civilisation for joining gold and silver for decorative purposes^19^ and further developed by Rev. J. T. Desaguliers, who adapted it for the cohesion of lead^20^. In his classic paper, dating back to 1725, the conditions leading to cohesion are documented in a way that is fully consistent with the existing models for cold bonding. Therefore, nearly 300 years later, these guiding principles are still valid. To enable bonding of two similar metals by CPW, the surface oxide layer on both sides of the joint line must first be removed or broken up mechanically by plastic deformation^21,22^. At the same time the oxide-free mating surfaces must be brought in intimate contact with one another on an atomic scale to allow the atoms across the interface to share their valence electrons^21–26^.

Because sharing of valence electrons does not involve diffusion of solute atoms across the faying interfaces, metallic bonding between two similar metals occurs instantaneously once the conditions for this to happen are reached. For cold bonding of dissimilar metals, the situation is different. For example, it follows from the binary Al-Cu phase diagram that both aluminium (Al) and copper (Cu) form solid solutions with each other at low and intermediate temperatures^27^. Hence, metallurgical bonding between these two metals can, in principle, occur by a mechanism involving interdiffusion of Al and Cu atoms. When the solid solubility limits are exceeded, different IMCs begin to precipitate. In most dissimilar metal joints, metallurgical bonding is achieved through IMC formation^28^. The type of IMC that forms will depend on the kinetics, but both Cu_9_Al_4_, CuAl and CuAl_2_ are examples of phases that have previously been observed in aluminium-copper welds^29–31^.

In cases where the joining operation leads to an increase in the interface temperature to between 300 and 400 °C, the thermal diffusivity of, for instance, Cu in Al is sufficiently high to facilitate interdiffusion and IMC formation, thus enabling adequate metallurgical bonding^32^. However, at room temperature (RT), the thermal diffusivity is far too low to promote long-range diffusion of Cu atoms across the interface, which makes it difficult to explain why cold bonding of Al to Cu remains feasible and even can be used for connecting aluminium busbars to copper terminals^22^. No characterisation techniques have been able to truly reveal the fundamental bonding mechanisms, even though some partial explanations can be found in the literature. In cold-welded Al-Cu samples produced using the equal channel angular extrusion process, scanning electron microscopy (SEM) has indicated that mechanical interlocking is the dominant factor contributing to the observed strength^33^. Beyond this, very little information is available in the scientific literature on the bonding mechanisms involved. Thus, the aim of the present study is to provide clarity on this matter.

The results demonstrate that metallurgical bonding between aluminium and copper at room temperature is enabled by the formation of excess vacancies during plastic deformation, which facilitates interdiffusion and the formation of intermetallic compounds. These findings establish excess vacancy generation as a key mechanism in cold bonding of dissimilar metals.

## Results and discussion

**Fig. 1:**
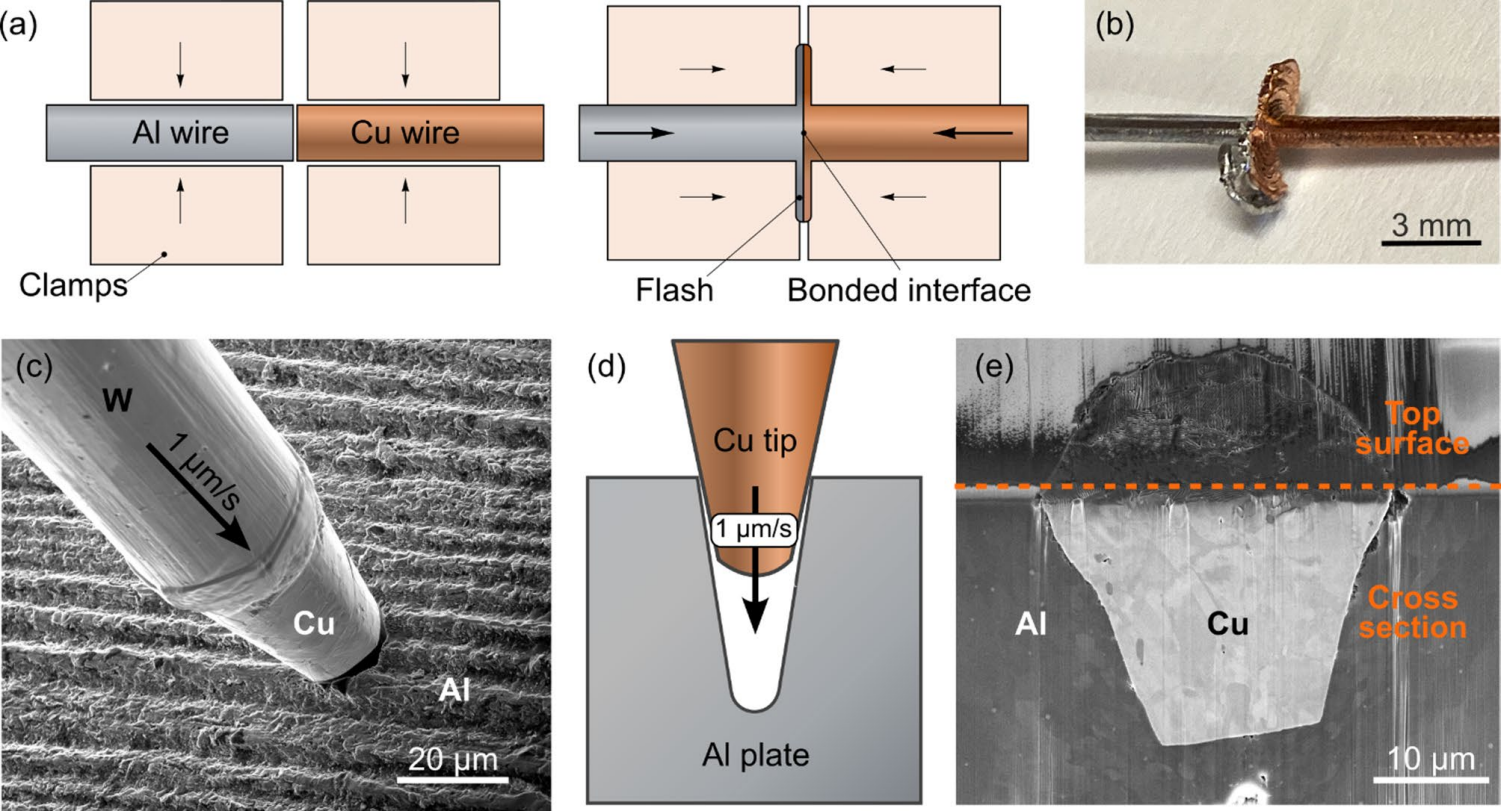
(**a**) Schematic view of the cold butt welding set-up with wires confined inside clamps. (**b**) Photograph of Cu and Al wires after cold butt welding. (**c**) SEM image showing the Cu needle positioned above the hole in the Al base metal prior to joining. (**d**) Schematic view of the microscale joining process. (**e**) Cross-sectional SEM image of the dissimilar metal joint after joining. Note that the orange dotted line marks the junction between the original top surface and the sputtered cross-section.

As a starting point for the investigation, two different cold pressure Al-Cu joints were selected. The first was produced using conventional butt welding of mm-sized wires in a cold welder machine. A schematic of the set-up employed for cold butt welding is shown in Fig. 1a. The two Cu and Al wires, each 1.4 mm in diameter, were confined inside clamps. Through manual and repeated operations, the wires were pressed against each other until bonding was achieved. The initial oxide and contamination layers on the cross-section surfaces of the wires were squeezed out through a gap in the confining clamps, forming flash, as shown in Fig. 1b. The second joint was produced using a novel technique, developed by the authors, for in-situ micro-joining of Cu to Al by shear deformation inside a focused ion beam (FIB) microscope^34,35^. Figure 1c–e show SEM images and a sketch of the cold welding process where a tapered Cu wire is pushed down into a premade hole in a soft Al base material. The tip of the tungsten micromanipulator needle of the FIB was replaced with Cu. This Cu tip was then pushed down into a hole in the soft Al base material by a speed of 1 µm/s. Further details about this methodology can be found in^35^. Since the average diameter of the tapered Cu wire is larger than the corresponding average diameter of the hole, the Cu and the Al alloys locally face large normal and shear forces while the Cu tip penetrates the hole. Even though surface oxide layers can be sputtered away by the ion beam in the high vacuum prior to welding, this was not performed in this case. However, the penetration of the hole by the larger Cu wire drags most of the surface oxides and contaminants down into the bottom of the hole, leaving a clean interface between the alloys.

Even though all experiments were conducted at RT, the resulting Al-Cu joints were mechanically strong. Tensile tests (Supplementary Fig. 1) demonstrate that the ultimate tensile strength of conventional butt welded joints is comparable to that of the soft, commercially pure aluminium base metal, with necking and fracture occurring in the Al wire away from the joint interface. Celotto et al.^36^ have previously shown that large plastic deformation accompanied by grain refinement and a change in crystallographic texture are involved in these cold butt-welding processes. However, for wires made of dissimilar metals, removal of interface oxides and large plastic deformation alone should not be sufficient to provide a strong joint. Usually, assistance from thermally driven interdiffusion of elements across the interface would also be necessary. Backscattered electron SEM images from a cross-section of a successful cold butt welded Al-Cu interface are shown in Supplementary Fig. 2. No porosity or mechanical interlocking can be observed along the Al-Cu interface. Not even IMCs, which typically indicate a strongly bonded interface, are observed along the interface at the maximum spatial resolution offered by SEM.

**Fig. 2:**
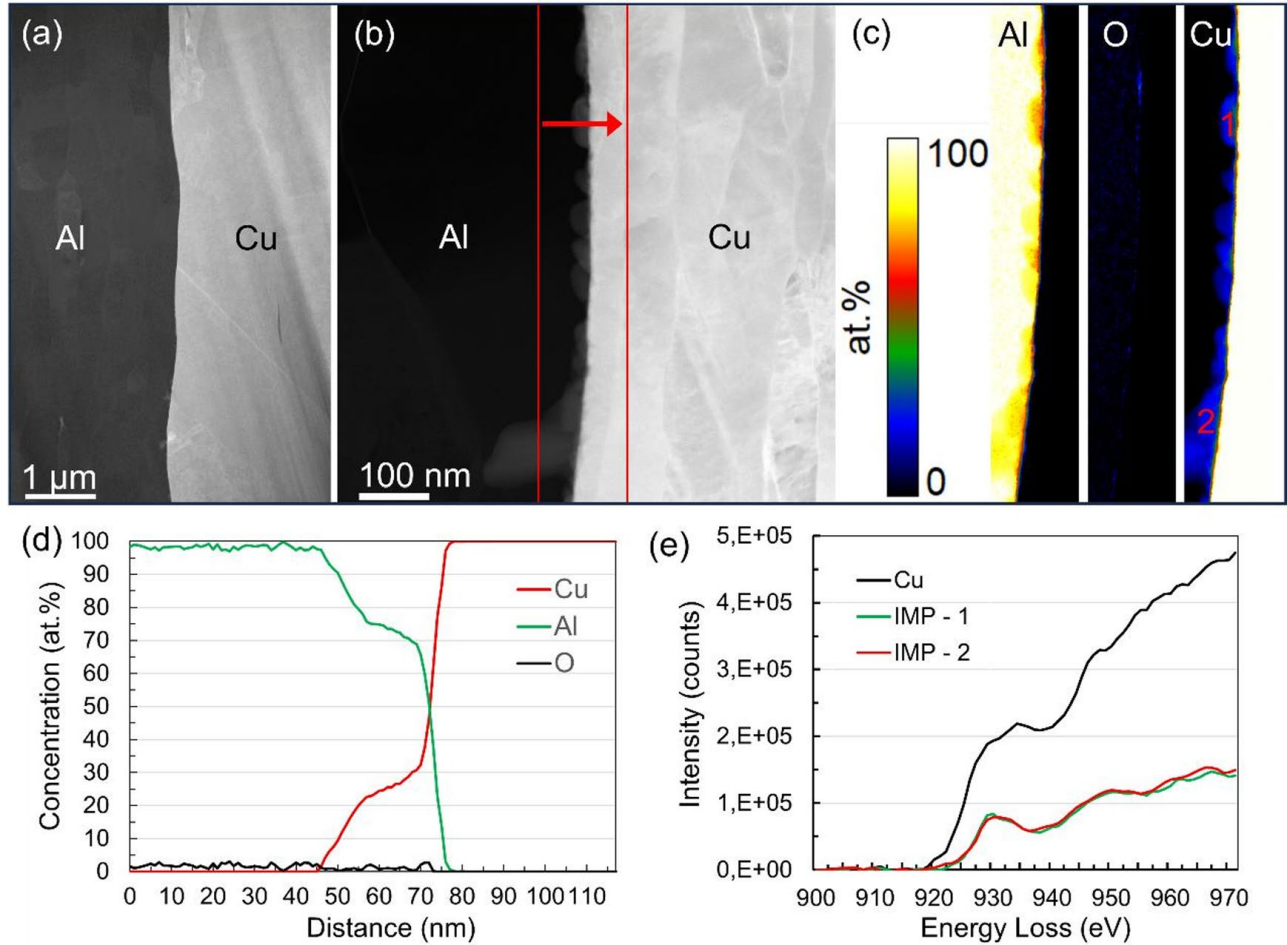
High angle annular dark field scanning transmission electron microscopy (HAADF STEM) images of the Al-Cu interface is shown in (**a**) and (**b**). Quantified Al, O and Cu maps, from the red framed area in (**b**), based on electron energy loss spectroscopy (EELS) mapping, are shown in (**c**). (**d**) Line profiles showing the chemical composition along the direction of the red arrow in (**b**). Background subtracted EEL Cu L_2,3_ edges from locations 1 and 2 in the Cu map and from the Cu bulk.

To understand the bonding mechanisms at the Al-Cu interface, beyond the resolution of SEM, cross-sectional TEM lamellae were prepared by FIB. A low-magnification high-angle annular dark field scanning transmission electron microscopy (HAADF STEM) image is shown in Fig. 2a. Similar to backscattered electron SEM images, only a sharp interface without IMCs is observed. At higher magnification, more details in the interface can be seen; the HAADF STEM image in Fig. 2b shows small nanoscopic islands of higher contrast et al.-side of the interface. Since the contrast has a strong atomic number dependency in HAADF STEM images, with heavy elements scattering more strongly to high angles, this image indicates that Cu has diffused across the interface into the Al. To further confirm cold diffusion of Cu into Al, the red framed region in Fig. 2b was mapped by electron energy loss spectroscopy (EELS). The only detectable elements in the EEL spectra were aluminium, oxygen and copper. Quantified maps (in at. %) with these elements are shown in Fig. 2c. The Cu map demonstrates that the high-contrast islands on the Al side of the interface in the HAADF STEM image are rich in Cu. A line profile that reveals the chemical composition along the direction of the red arrow in Fig. 2b is shown in Fig. 2d. At this location, we see that Cu has diffused up to 25 nm into the Al. Along the entire macroscopic interface, small islands of intermetallic Cu_x_Al_y_ have developed on the Al side of the interface. Moreover, the original oxides at the interface were removed, while the small amount detected comes from the oxidised top surface of the Al side of the sample.

The chemical composition, i.e. the values of x and y in Cu_x_Al_y_, cannot easily be extracted from the quantified element maps or the line profiles, since the small IMC islands do not extend through the full thickness of the TEM lamella. Hence, both the IMC islands and the surrounding Al matrix contribute to the chemical composition at the projected location of the islands. This challenge, to extract the real composition of the nanoscopic IMC islands, highlights a general challenge in TEM: Aberration-corrected TEM data can potentially provide images and spectroscopy information with atomic resolution in the plane perpendicular to the electron beam, but must contend with the projected averaging along the direction of the electron beam. Hypothetically, this further means that we cannot be sure that the claimed islands are IMCs. It is also a possibility that the Al-Cu interface has a very strong interface topography. What looks like nanoscopic IMC islands may, in fact, be pure Cu islands penetrating into the Al. Hence, in projection, overlap between pure Al and pure Cu can mimic the formation of IMCs. This would further mean that there is no diffusion of Cu across the interface to facilitate a mechanically strong joint. To exclude that the argued interdiffusion with formation of IMC islands is not a simple overlap of the pure metal alloys, the Cu L_2,3_ edge from the EELS data is shown in Fig. 2e. The black line shows the L_2,3_ edge acquired in the bulk of Cu far away from the interface. The red and green lines are from two of the Cu-rich islands, as labelled in the Cu map in Fig. 2c. The L_2,3_ edges from the assumed IMCs are significantly different from the Cu reference spectrum. The fine structure, and particularly the extended near edge fine structure, of the edges from the islands is different from the Cu alloy reference. Both the coordination and chemical environment around each Cu atom have changed compared to the Cu reference. These changes in the edge fine structure prove that the signal comes from a different compound than the face-centred cubic (fcc) Cu and can only be explained by the formation of IMCs, where the symmetry and neighbours surrounding each Cu atom have changed from a simple fcc Cu structure.

**Fig. 3:**
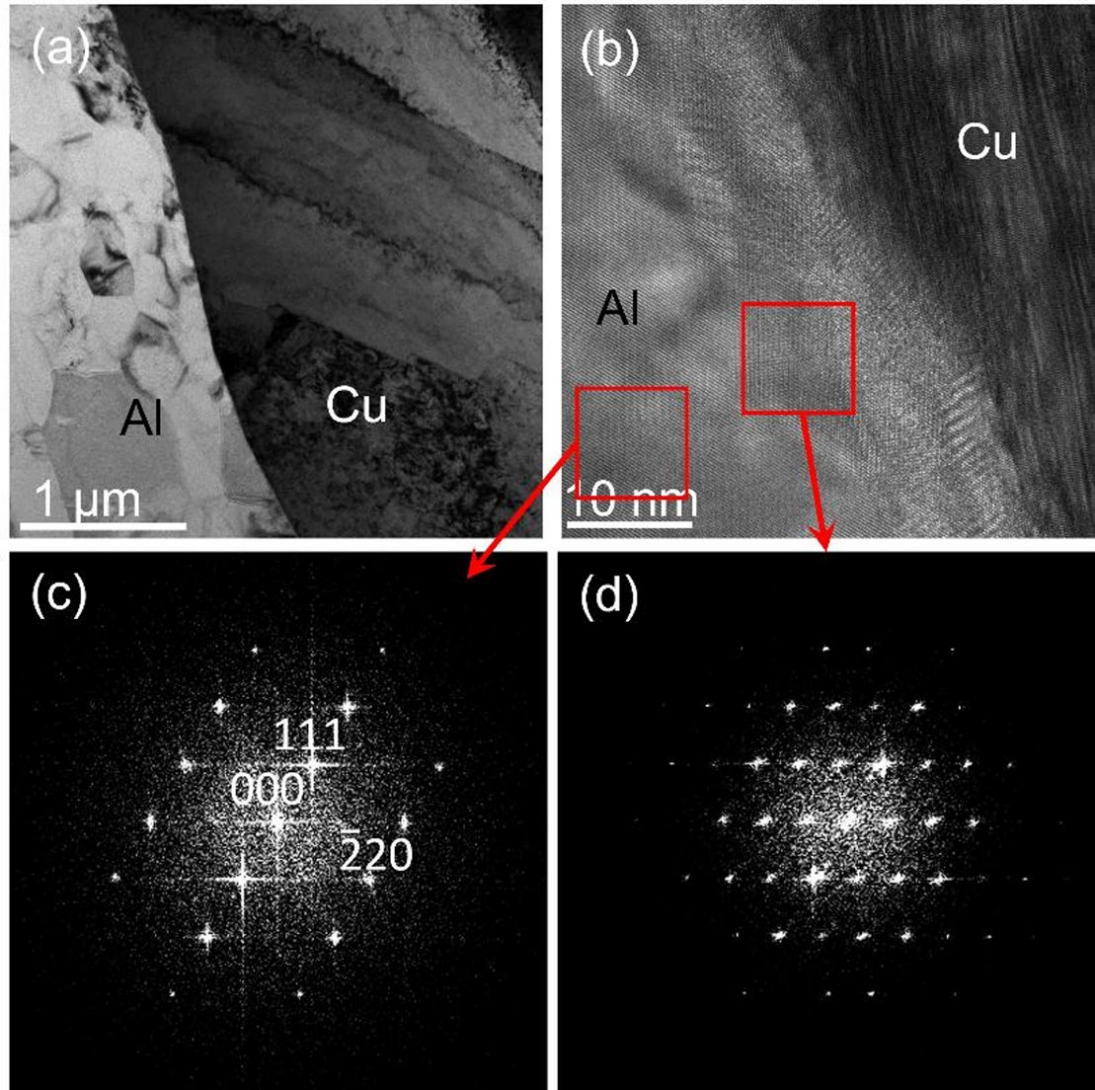
(**a**) Low and (**b**) high magnification bright field TEM images from the Al-Cu interface. Fourier transforms from the two, red squared frames in (**b**) are shown in (**c**) and (**d**).

Further evidence supporting the formation of IMCs due to the diffusion of Cu into Al is presented in Fig. 3. The low magnification, bright field (BF) TEM image in Fig. 3a displays a seemingly clean and abrupt Al-Cu interface. However, the high-resolution image in Fig. 3b reveals an interfacial layer with a different contrast compared to the neighbouring Al and Cu grains. The aluminium grain close to the interface is oriented on a [110] zone axis. This orientation is demonstrated in the fast Fourier transform (FFT) in Fig. 3c. Within the interface layer, the FFT shown in Fig. 3d reveals a crystallographic unit cell significantly larger than those of either pure Al or Cu. The copper grain on the other side of the interface is away from any high symmetry orientation, and the FFT inside the interface layer is inconsistent with a Moiré pattern arising from simple crystal overlap of Al and Cu. Hence, these observations strongly support that the islands previously identified in Fig. 2 are indeed IMCs formed during the cold-welding process.

Grain refinement is observed near the Al–Cu interface, especially on the aluminium side, where the grain size is reduced from ~ 10 µm to sub-micrometre scale (Fig. 3a). While this increases the density of grain boundaries, their contribution to diffusion and vacancy annihilation is expected to be limited at room temperature. Moreover, the spatial distribution of the intermetallic CuₓAlᵧ islands does not correlate with the grain boundaries, suggesting that grain boundary diffusion is not the dominant mechanism. These observations support the conclusion that excess vacancies generated by plastic deformation play a more significant role in interfacial diffusion and intermetallic formation during cold bonding.

**Fig. 4:**
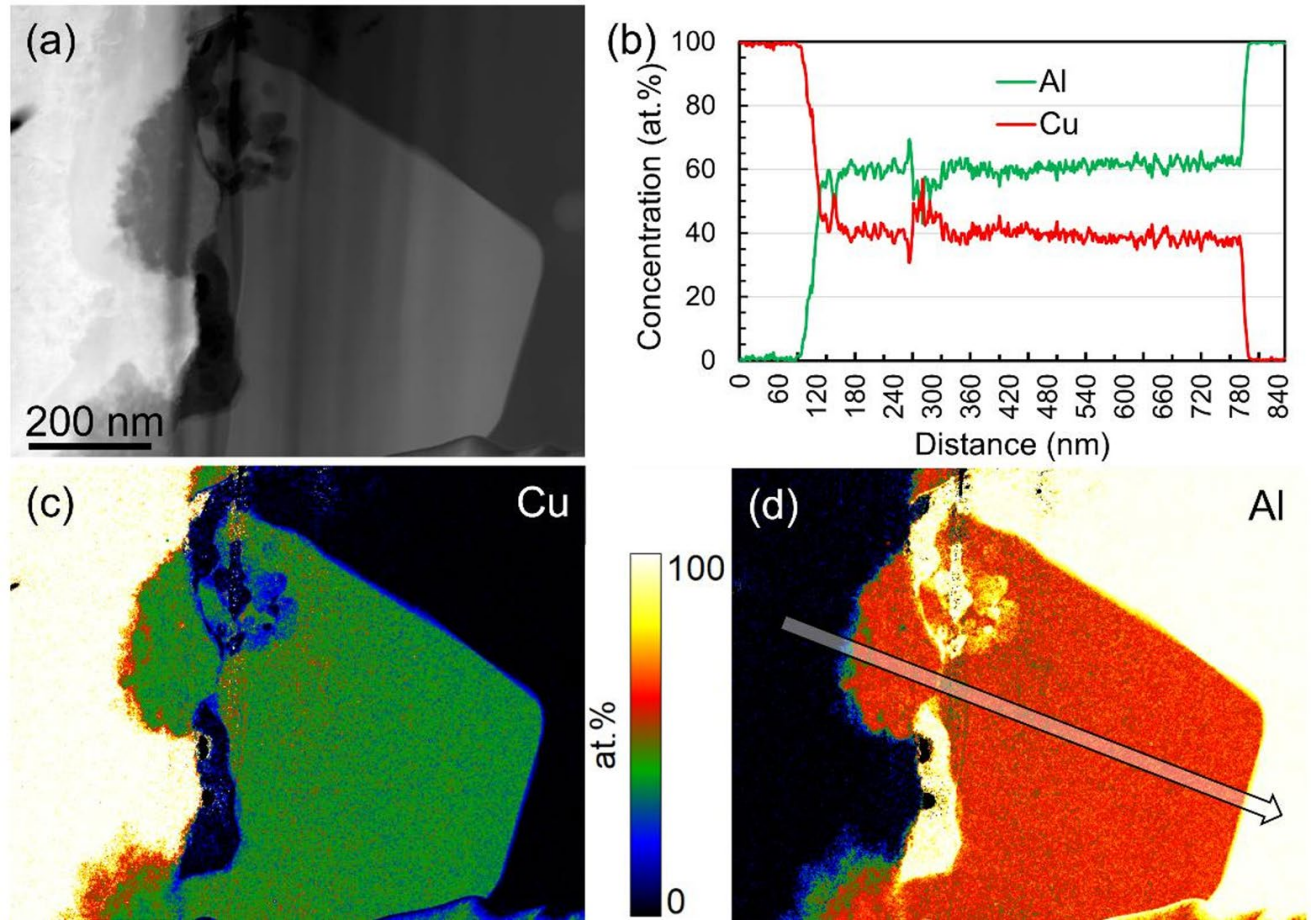
(**a**) High angle annular dark field scanning transmission electron microscopy image from the Al-Cu interface. Quantified Cu and Al maps, based on electron energy loss spectroscopy, from the same region is shown in (**c**) and (**d**) respectively. A line profile showing the chemical composition along the direction of superimposed arrow in the Al map is shown in (**b**).

Several TEM lamellae were also collected along the interface from the in-situ micro joints between the Cu tip and the Al base material. A HAADF STEM image, along with quantified Cu and Al maps from a region subjected to the highest plastic strain, is shown in Fig. 4. An intermetallic layer over 0.5 µm thick has formed at the interface. This IMC has primarily resulted from Cu diffusion into Al, leading to the growth of a large, facetted Cu_x_Al_y_ grain. Unlike the case of cold butt welding, Al has also diffused into the Cu, resulting in the formation of IMC particles on the Cu side. However, Al diffusion remains significantly more limited than Cu diffusion. As a result, the IMCs on the Cu side are smaller and lack well-defined facets toward the Cu matrix. The line profile in Fig. 4b indicates that the formed IMC is Al-rich. The exact chemical and crystallographic nature of the IMC was not determined, as it was considered non-critical to identifying the fundamental mechanisms underlying cold-weld formation.

The final and most important question is how IMCs can form at temperatures close to room temperature. The mechanical integrity and strength observed at the joint interfaces after both cold butt welding and microscale welding in the FIB are clearly due to diffusion across the Al-Cu interface, leading to IMCs formation. However, the available thermal energy is far too low to support conventional diffusion in either case. The tens of nm diffusion in the case of cold butt welding, and up to 0.5 µm diffusion in the micro-joining set-up, must therefore be due to a different mechanism than temperature activated diffusion. The answer to this question can be found in the early work by Militzer et al.^37^ and more recently in the work of Robson^38^. In addition to thermally activated diffusion, defects in the form of vacancies and dislocations can significantly enhance diffusion. In the present cold pressure joints, the plastic strain introduced at low temperatures increases the vacancy concentration by several orders of magnitude relative to the equilibrium thermal concentration. Based on the model by Militzer, a simplified analytical expression describing the production rate of excess vacancies can be written as:

1 $$\frac{d{c}_{ex}(t)}{dt}=(M+T)\frac{d\epsilon }{dt}-{D}_{v}\left(D\left(t\right)+G\right){c}_{ex}(t)$$

**Fig. 5:**
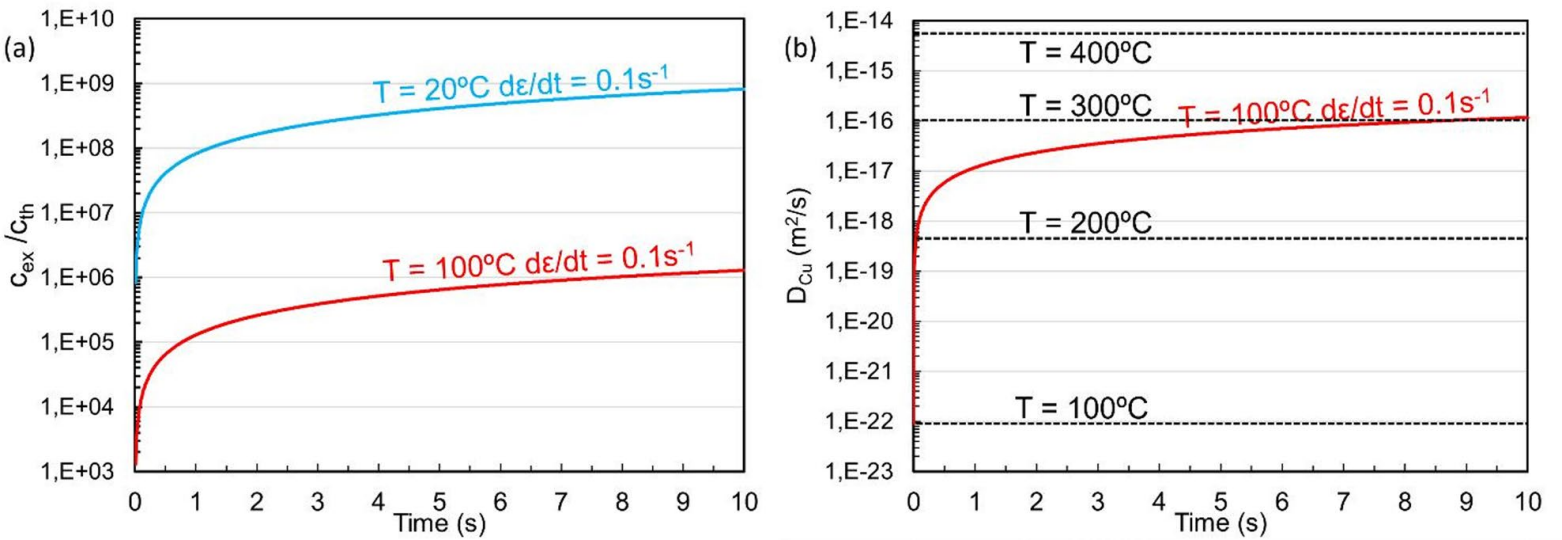
(**a**) Creation of excess vacancies compared to thermal vacancies at room temperature (blue line) and 100 °C (red line) at 0.1 strain rate. In (**b**), the red line shows the effect of excess vacancies on the diffusion coefficient of Cu in Al at 100 °C for a strain rate of 0.1. The dashed lines show the thermal contribution to the diffusion constant of Cu in Al at various temperatures, as described by Supp. Eq. 4 in the Supplementary Information 1.

*M* represents the mechanical and *T* the thermal contribution to vacancy generation. These two terms contribute to the formation of excess vacancies in proportion to the strain rate. Additionally, there are dislocation (*D*) and grain boundary (*G*) related terms linked to the annihilation of excess vacancies. Due to increasing strain and plastic deformation, the dislocation term *D* also has a time dependence, since the dislocation density increases as a function of time and applied strain. Furthermore, the annihilation terms are proportional to the vacancy diffusion coefficient *D*_*v*_, which is temperature dependent. For our cold welding examples, the temperature is expected to remain below 100 °C. After cold butt welding of the Cu and Al wires, no significant temperature increase was observed on the surface of the bonded wires. Considering the high thermal conductivity of both metals, the bonded interface is not expected to reach temperatures above 100 °C. In Fig. 5a the number of excess vacancies compared to thermal vacancies is plotted as a function of time for a strain rate of 0.1 s^-1^ at 20 °C and 100 °C, using Eq. (1). Full calculation details and term definitions are provided in Supplementary Methods 1. Material constants for aluminium are taken from^38^. A strain rate of 0.1 s^-1^ and total strain of 1 (after 10 s) are conservative values for both joining techniques applied in this work. For the cold butt welded wires, a macroscopic strain of more than 1 is typically needed for successful bonding^36^. At the interface between the two dissimilar metals, the strain rate is locally higher than the average over a broader reference volume. Even with the conservative numbers used for the plots in Fig. 5a, the strain rates during the welding process increase the vacancy concentration by more than 6 orders of magnitude at 100 °C and almost nine orders of magnitude at room temperature.

The diffusion of Cu in Al, *D*_*Cu*_, scales with the concentration of vacancies. In Fig. 5b we therefore observe that mechanically generated excess vacancies result in a diffusion rate equivalent to thermally activated diffusion at temperatures more than 200 °C higher than the welding temperature. Considering that the Cu-Al interface region for both of the presented cold welding set-ups generally experiences a strain rate and a total strain significantly higher than 0.1 s^-1^ and 1, respectively, the diffusion rate at the bonded interface is likely to exceed the values simulated in Fig. 5b.

With regard to the diffusion rates are needed to create intermetallic layers at Cu-Al interfaces, hybrid metal extrusion & bonding (HYB) serves as a good reference. Sørhaug^39^ and Bergh^18^ have both shown that HYB generates a 200–300 nm thick IMC at the Cu-Al interface. This intermetallic layer is formed by thermally activated diffusion at temperatures in the range 400–500 °C, lasting for a few seconds. In the present welds, a much thinner IMC is generally observed. A diffusion constant below what is observed from thermal diffusion at 400 °C is therefore expected. The total time to form the IMC can also be significantly different between HYB and the present cold welding techniques. During HYB, IMCs are formed only during the brief period when the temperature is high enough to support diffusion. In the temperature regime below 100 °C, Robson^38^ has shown that the excess vacancy concentration decays very slowly, and diffusion can therefore continue for a significant period even after the strain rate drops to zero. This further implies that IMCs can form over a longer timescale extending beyond the actual bonding event.

High strain rates and large plastic deformations are also involved in HYB, as well as other solid-state welding techniques, such as friction stir welding. However, the excess vacancies generated in these techniques still do not govern IMC formation, due to the high temperature. The temperature dependent annihilation terms in Eq. (1) balance the generation terms and remove the vacancies at the same rate as they are created. As the temperature decreases behind the weld line in these processes, the excess vacancy concentration also decreases accordingly.

## Outlook

Even though cold bonding of dissimilar metal alloys has occasionally been demonstrated in the literature, few commercial applications of room temperature welding are observed. One of the main bottlenecks that has hindered the widespread utilisation of cold welding is the lack of fundamental understanding of successful bonding mechanisms. This lack of understanding has made it impossible to tailor and design successful welding set-ups. In the present work, we have demonstrated that, in addition to the removal of surface oxides and contaminants, the formation of a high concentration of excess vacancies is a key enabling factor in a cold bonding set-up. The excess vacancy concentration can be tuned and controlled through control of strain, strain rate and temperature. Simple existing models can further be used to predict diffusion, and hence the formation of intermetallic compounds and the resulting bond strength at the welded interface. This new insight may pave the way for replacing many of today’s friction and fusion-based techniques with cold welding methods, which offer the advantages of much thinner intermetallic layers at the interface, the absence of heat-affected zones, and low energy input.

## Methods

### Cold butt welding (CBW)

Cold Butt Welding (CBW) of Electrolytic Tough Pitch copper (ETP Cu) and commercially pure AA1070 aluminium was conducted using a manual BWE Ltd CW2E Cold Welder machine, commonly employed for repairing electrical copper wires while being produced. The process consists of placing two wires into the machine clamps, aligning their ends and applying repeated lever thrusts. The lever operates the dies, which grip the wires and bring them in contact while increasing the material supply at the contact zone. This enables bonding to occur and excess material to be expelled as a brittle flash. More details about the technique are thoroughly reported in a previous work^36^.

### In situ mechanical testing of CBW joints

In-situ tensile testing of the CBW Al-Cu joints was performed using a Kammrath Weiss GmbH DDs-3 testing module positioned inside a Thermo Fisher QUANTA 650 field emission gun (FEG) SEM. To confine the fracture location within a selected region across the interface, the central part of the welded specimens was machined to a gauge length of approximately 10 mm and a diameter of 1.0 mm (the initial diameter of the wires was 1.4 mm). The bonded interface was positioned at the centre of the gauge section. A schematic illustration of specimens’ geometry is shown in Supplementary Fig. 3. Supplementary Fig. 4 shows SEM images of the Al-Cu joints before and after tensile testing.

### Focused ion beam microscale cold welding

The microscale cold welding was performed as described in detail elsewhere^34,35,40^. Holes in the AA1070 base material were made with a xenon (Xe)-based plasma(P)FIB (Helios 5) to avoid Ga implantation into the Al alloy. The ETP Cu needle was prepared inside a Helios G4 UX Ga-FIB, while the cold welding was performed inside a Helios Nanolab 600. All FIBs were from Thermo Fisher Scientific.

### Characterisation of the micro-joints

Following the joining process, cross-sections running through the centre of the joints (as shown in Fig. 1e) were prepared with an Xe-based Helios 5 PFIB. Cross-section TEM lamellae were further prepared by a Helios G4 UX according to the following procedure: Carbon-based protection layers were first deposited on top of the regions of interest. The first part of the protection layer was made by electron beam-assisted deposition to avoid any ion beam damage to the Cu-Al joint. The lamellae were transferred and mounted as flags to Cu FIB lift-out grids. All coarse thinning was further done with a 30 kV acceleration voltage for the ions. Final thinning was first done at 5 kV and then at 2 kV to minimise surface damage on either side of the lamellae.

TEM was performed with a double spherical aberration corrected cold FEG Jeol ARM 200 FC, operated at 200 kV. This instrument is equipped with a GIF Quantum ER for electron energy loss spectroscopy (EELS). Simultaneous EDS and dual-EELS spectrum imaging were performed in scanning transmission electron microscopy (STEM) mode. The zero-loss peak in the low-loss spectra in EELS was used to calibrate the energy scale in every pixel of a map, and to transfer this energy calibration to the high-loss spectra.

## Supplementary Information

Below is the link to the electronic supplementary material.

Supplementary Material 1

## Data Availability

The authors declare that the main data Supplementary the findings of this study are available within the article and its Supplementary Information files. Extra data are available from the corresponding author upon request.
