# Supplementary material for: Cold bonding of aluminium to copper by deformation-enhanced diffusion
Source: Sci Rep. 2025 Nov 24;15:41656. doi: 10.1038/s41598-025-25620-1 (PMC12645032; doi:10.1038/s41598-025-25620-1)
Supplement: Supplementary file 1 — Supplementary Material 1 [file 41598_2025_25620_MOESM1_ESM.pdf]

Supporting material

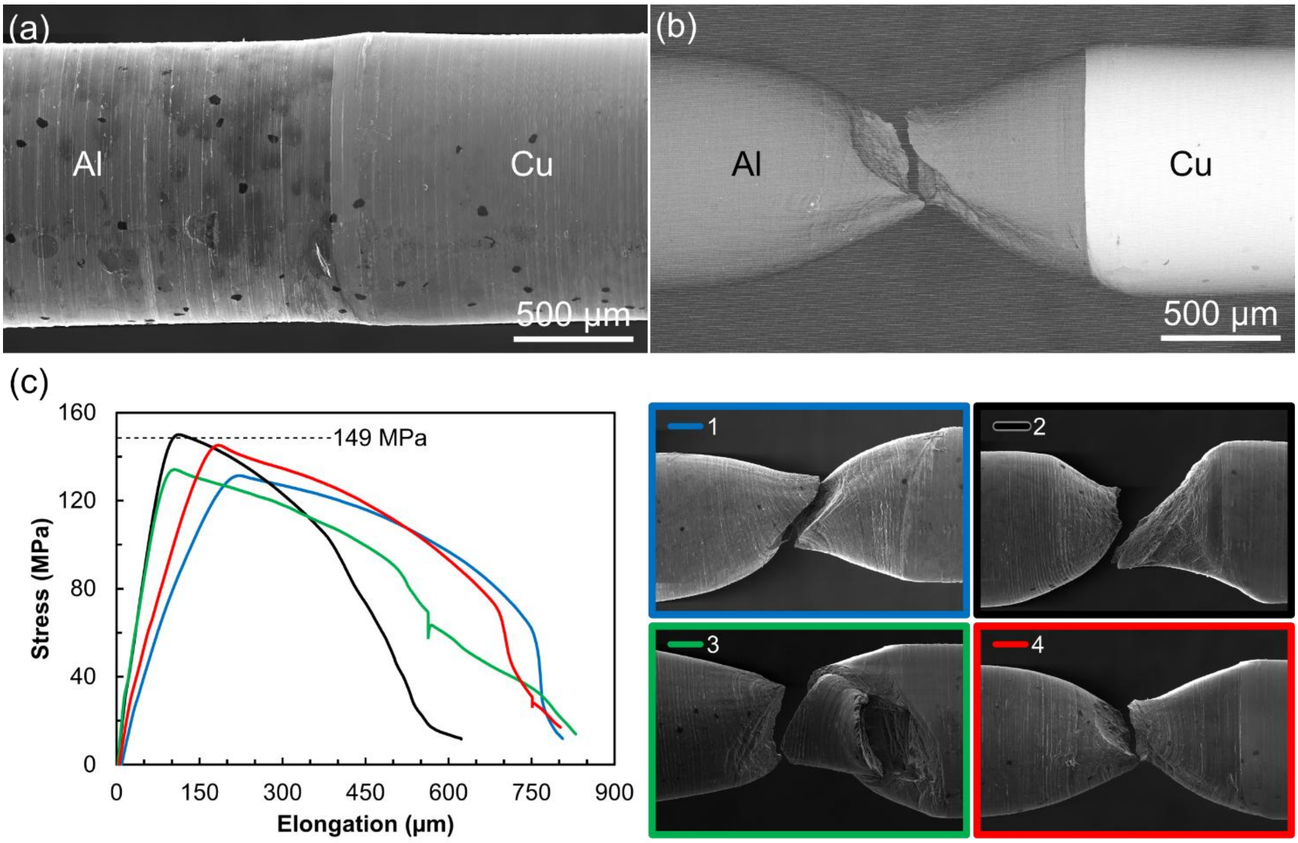

Supporting Fig. 1 *(a) Secondary electron and (b) backscattered electron images of the Al-Cu cold butt welded joint prior to and after tensile testing, respectively. The results of tensile testing of different joints are shown in (c). The dashed line, at 149 MPa, shows the ultimate tensile strength of the soft Al wire prior to any welding. Legend is given on the side by showing the fracture mechanisms of each respective specimen.*

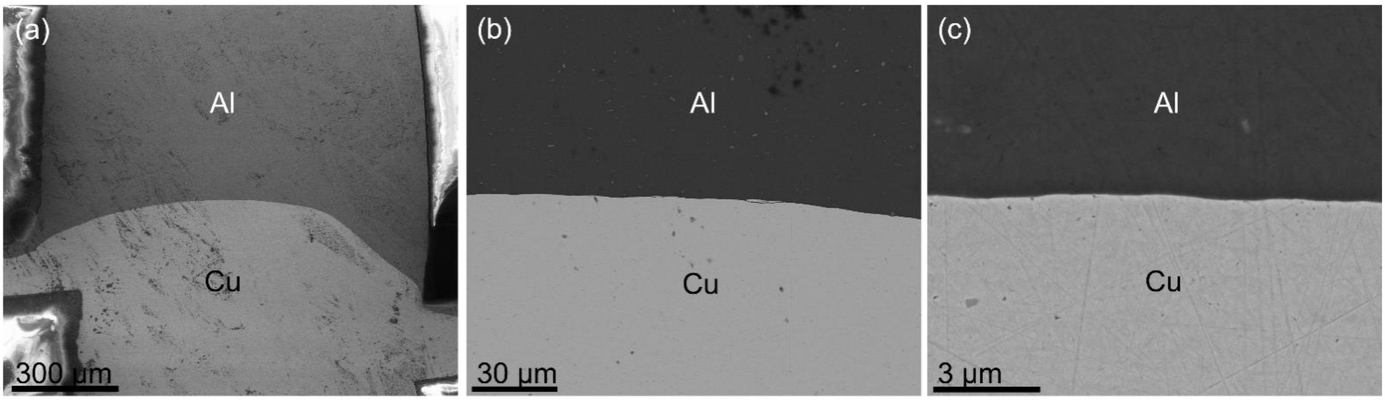

Supporting Fig. 2 *Backscattered electron images of a cross-section of an Al-Cu cold butt welded joint. The SEM images show the Al-Cu interface at progressively higher magnification.*

Supporting information 1

The production rate of excess vacancies, c_{ex}, as a function of strain rate, $\dot{\epsilon}$, is taken from the work of Militzer [1] and Robson [2] and is given by the equation:

$$\frac{dc_{ex}}{dt} = \left(\chi\frac{\sigma\Omega_0}{Q_f}\dot{\epsilon} + \zeta\frac{c_j\Omega_0}{4b^3}\dot{\epsilon}\right) - \left(\frac{D_v\rho}{\kappa^2} + \frac{D_v}{L^2}\right)c_{ex} \qquad \text{Supp. Eq. (1)}$$

The table below gives the numbers and material constants that was used to make Fig. 5(a) in the main document:

Parameter	Meaning of parameter	Value
χ	Total amount of energy introduced during the welding process that is used to create excess vacancies	*0.1*
Ω_0	Volume of a single aluminium atom	$1.66 \times 10^{-29}\ m^3$
σ	Flow stress of AA1070 aluminium alloy	*120 MPa*
Q_f	Vacancy formation energy	*0.76 eV*
$\dot{\epsilon}$	Strain rate	*0.1*
ζ	Neutralization effect caused by that thermal jogs can both emit and absorb vacancies	$= 0.5 - \zeta_0 c_j$
c_j	Concentration of thermal jogs	$= exp(-E_j/kT)$
E_j	Formation energy of screw dislocations	
b	Burgers vector	$2.9 \times 10^{-10}\ m$
D_v	Vacancy diffusion coefficient	$= D_{v0} exp(-Q_m/kT)$
D_{v_0}	Pre-factor for vacancy diffusion	$10^{-5}\ m^2s^{-1}$
Q_m	Activation energy for vacancy diffusion	*0.83 eV*
ρ	Dislocation density	*See below*
ρ_s	Steady-state dislocation density during deformation	$5 \times 10^{14}\ m^{-2}$
$\rho(0)$	Dislocation density prior to cold welding	$10^{10}\ m^{-2}$
κ	Dislocation arrangement	*1*
L	Average grain diameter	$10^{-6}\ m$
β_0	Material constant	*500 MPa*
D_{s_0}	Pre-factor for diffusion of solute Cu in the Al lattice	$2.9 \times 10^{-5}\ m^{-2}s^{-1}$
Q_s	Activation energy for diffusion of solute Cu in Al	$12\,500\ Jmol^{-1}$

For simplicity, the production of vacancies due to thermal jogs was set to zero. This was done since the concentration of thermal jogs, equal to exp(-E_j/kT), is extremely low at room temperature, and neglectable compared to the mechanical term.

The dislocation density, ρ, is given by the following expression:

$$\rho(t) = \rho_s - (\rho_s - \rho(0))\exp\left(\frac{\beta_0 \dot{\epsilon}}{\sigma} t\right) \qquad \text{Supp. Eq. (2)}$$

The diffusion coefficient of copper in aluminium, as shown in Fig. 5(b), was plotted based on the following equation:

$$D_{Cu} = D\left(1 + \frac{c_{ex}}{c_{th}}\right) \qquad \text{Supp. Eq. (3)}$$

In the expression above, diffusion along dislocations and grain boundaries was omitted. D is the thermal diffusion coefficient for copper in aluminium and was calculated based on the following expression:

$$D = D_{s_0} exp(-Q_s/RT) \qquad \text{Supp. Eq. (4)}$$

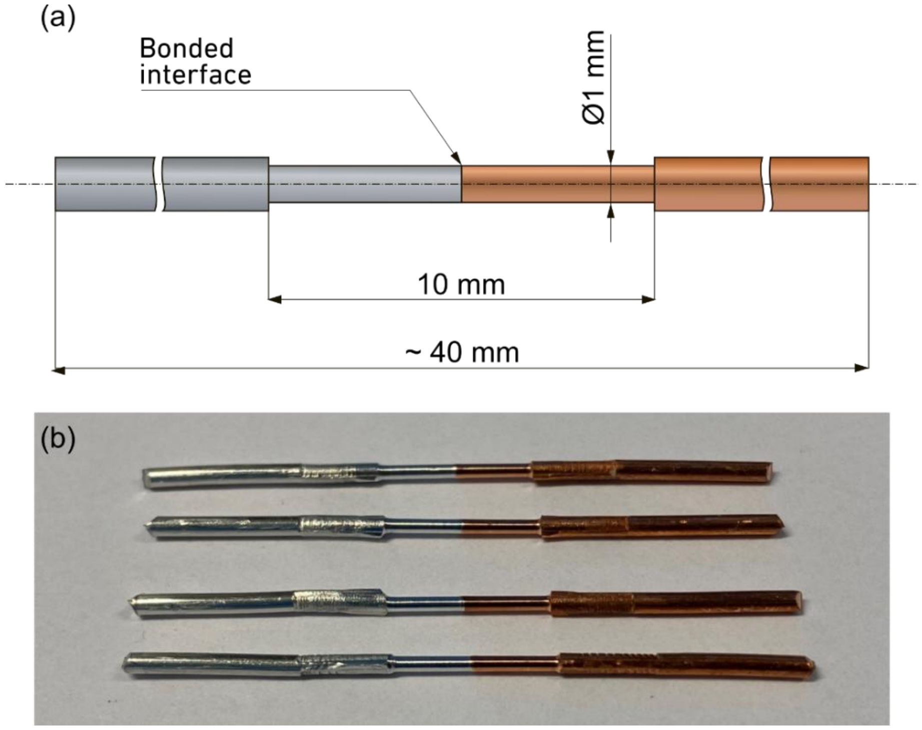

Supporting Fig. 3 *Illustration of the geometry and dimensions (a) and picture (b) of specimens used for in-situ tensile testing.*

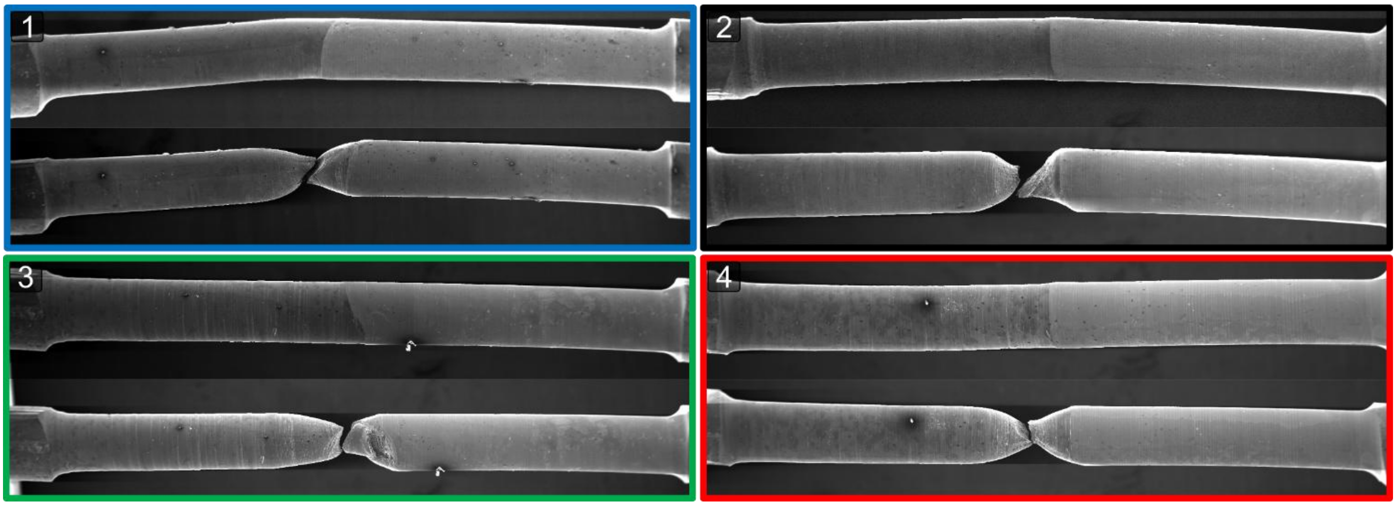

Supporting Fig. 4 *SEM images of the CBW specimens before and after tensile testing. Aluminium is on the left side, and copper is on the right side. Fracture always occurs in the weakest material and originates from the interface only in case of bonding defects (specimen no.3)*

Supporting references:

[1]. M. Militzer, W.P. Sun, J.J. Jonas: Modelling the effect of deformation-induced vacancies on segregation and precipitation, Acta Metall. Mater. Vol. 42 (1994) pp. 133-141.

[2]. J.D. Robson: Deformation Enhanced Diffusion in Aluminium Alloys, Metallurgical and Materials Transactions A, Vol. 51A, (2020), pp. 5401- 5413.
